# Genome-wide identification and characterization of Ethylene-Insensitive 3 (*EIN3/EIL*) gene family in *Camellia oleifera*

**DOI:** 10.1371/journal.pone.0324651

**Published:** 2025-05-23

**Authors:** Shihang Huang, Fang Li, Caiqin Li, Xiaobei Li, Qiuling Pan, Yongquan Li, Wenpei Song, Juan Li

**Affiliations:** College of Horticulture and Landscape Architecture, Zhongkai University of Agriculture and Engineering, Guangzhou, Guangdong, China; University of Massachusetts Amherst, UNITED STATES OF AMERICA

## Abstract

*Camellia oleifera*, a vital woody oil crop in China, suffers substantial yield losses due to frequent physiological fruit abscission during cultivation. Ethylene signaling, mediated by Ethylene-Insensitive3/Ethylene-Insensitive 3-like (EIN3/EIL) transcription factors encoded by a multigene family, plays a pivotal role in plant organ abscission. However, the EIN3/EIL family remains understudied in *C. oleifera*. Here, genome-wide analysis identified four *CoEIL* genes encoding proteins with conserved EIN3 domains. Phylogenetic classification grouped these proteins into Group A and Group B, revealing evolutionary proximity between *C. oleifera* and tea (*Camellia sinensis*). *Cis*-acting regulatory element analysis implicated *CoEIL* genes in hormone responsiveness and stress adaptation. Quantitative real-time PCR analysis was performed to investigate the expression patterns of these *CoEIL* genes in the fruit abscission zone following ethephon and brassinolide treatments. This study elucidates the genetic architecture and functional divergence of *CoEIL* genes, laying the foundation for exploring molecular mechanisms of abscission in *C. oleifera*.

## Introduction

*Camellia oleifera* Abel. (commonly termed oil-camellia), a woody plant extensively cultivated in southern China, ranks among the world’s four most economically significant perennial oil crops, alongside oil palm (*Elaeis guineensis*), olive (*Olea europaea*), and coconut (*Cocos nucifera*) [1]. Oil-camelia produces a premium edible oil, often referred to as “Oriental olive oil”, owing to its high nutritional value. The oil is characterized by elevated levels of unsaturated fatty acids, including approximately 75–83% oleic acid and 7–13% linoleic acid, which are associated with reduced risks of cardiovascular diseases [2]. Despite its agricultural importance, excessive fruit abscission remains a critical constraint on *C. oleifera* yield. Three distinct abscission phases occur during fruit development [3]: 1. Floral abscission: This initial phase begins at the termination of full female flowering and persists for roughly four months. 2. Juvenile fruit abscission: Occurring between April to July, this stage is predominantly driven by fertilization failure and embryonic developmental abnormalities. 3. Preharvest abscission: Primarily induced by biotic stressors (e.g., pathogens, pests) and mechanical injury, this phase directly impacts final harvest yields. However, the molecular and physiological mechanism governing fruit abscission in *C. oleifera* remain poorly elucidated.

Fruit abscission in plants is a genetically regulated process that facilitates the shedding of unfertilized, damaged, infected, nutritionally deficient, mature, or senescent fruits [4]. This phenomenon is typically initiated by environmental stressors or developmental cues through the activation of cell separation mechanisms within specialized tissues termed abscission zones [5–6]. Phytohormonal regulation, particularly involving ethylene, plays a dominant role in controlling this process [7–9]. Experimental evidence demonstrates that exogenous application of ethephon (an ethylene-releasing compound) induces premature flower or fruit abscission in multiple economically significant crops, including grape (*Vitis vinifera*), mango (*Mangifera indica*), litchi (*Litchi chinensis*), peach (*Prunus persica*), and oil palm [10–14]. Conversely, suppression of ethylene biosynthesis via aminoethoxyvinylglycine (AVG), a competitive inhibitor, enhances fruit retention rates in apple (*Malus × domestica*) orchards [15–16].

In ethylene signaling, ethylene-insensitive3/ethylene-insensitive3-like (EIN3/EIL) transcription factors serve as central transcriptional regulators that activate downstream gene expression cascades by directly modulating ethylene-responsive genes [17–18]. EIN3/EIL homologs have been characterized across diverse plant species, including *Arabidopsis thaliana* [19–20], litchi [21], tomato (*Solanum lycopersicum*) [22–23], apple [24], rice (*Oryza sativa*) [25–27], rubber tree (*Hevea brasiliensis*) [28], and poplar (*Populus trichocarpa*) [29]. Structurally, EIN3/EIL proteins localize to the nucleus and exhibit conserved N-terminal motifs critical for function, including acidic amino acid clusters, five basic domains (I-V), and proline-rich sequences [19,30]. These transcription factors regulate key physiological processes such as fruit ripening [22,31,32], leaf senescence [33–34], and abiotic/biotic stress acclimation [35–37]. Moreover, *EIN3/EIL* genes mediate hormonal cross-talk between ethylene and other phytohormones, including salicylic acid, jasmonic acid, and brassinosteroids, thereby integrating multiple signaling networks [38–40].

This study aimed to systematically identify and characterize the *EIN3/EIL* transcription factor family genes within the *C. oleifera* genome, and to investigate the expression dynamics of *EIN3/EIL* genes during fruit abscission. Utilizing genomic data, we conducted comprehensive genome-wide screening to identify all *C. oleifera EIN3/EIL* genes (*CoEIL* genes). Phylogenetic relationships among CoEIL proteins were resolved through comparative analysis with homologs from multiple species, and temporal expression profiles of these genes were quantified during distinct abscission stages. These findings establish a conceptual framework for elucidating the roles of *CoEIL* genes and the molecular mechanisms underlying fruit abscission in *C. oleifera*.

## Materials and methods

### Identification and sequence analysis of *CoEIL* family genes

Coding DNA sequences and corresponding protein sequences were retrieved from the published *C. oleifera* genome [41]. To identify homologous EIN3/EIL proteins, *A. thaliana* EIN3/EIL protein sequences, obtained from The *Arabidopsis* Information Resource (TAIR) database (https://www.arabidopsis.org/) served as query templates for a BLASTP search against the annotated *C. oleifera* proteome using TBtools [42] with default parameters (E-value = 1 × 10^-5^, maximum hits = 500, alignments = 250, and threads = 2). Candidate CoEIL sequences were refined through reciprocal BLAST and validated for the presence of the conserved EIN3 domain (PF04873) via NCBI CDD (Conserved Domains Database, http://www.ncbi.nlm.nih.gov/Structure/cdd/wrpsb.cgi), Pfam (http://pfam.sanger.ac.uk/), and SMART databases (the HMMER-based Simple Modular Architecture Research Tool, http://smart.embl-heidelberg.de/). Physicochemical properties of CoEIL proteins, including molecular weight and isoelectric point, were calculated using the ExPASy ProtParam tool (http://web.expasy.org/compute_pi/) [43]. Subcellular localization predictions were generated using Cell-PLoc v2.0 (http://www.csbio.sjtu.edu.cn/bioinf/Cell-PLoc-2/) [44].

### Multiple sequence alignment and phylogenetic analysis

Multiple sequence alignment of EIN3/EIL homologs from *C. oleifera*, *A. thaliana*, and litchi was performed using ClustalX v1.83 [45], with subsequent visualization in GeneDoc v2.7 [46]. For phylogenetic reconstruction, EIN3/EIL protein sequences from 12 phylogenetically diverse species were curated from the NCBI (https://www.ncbi.nlm.nih.gov/guide/) and Phytozome (https://phytozome.jgi.doe.gov/pz/portal.html) databases. Alignments generated via ClustalX were used to reconstruct a Neighbor-Joining phylogenetic tree in MEGA 7.0 [47], employing the Poisson model, 1,000 bootstrap iterations, and complete-deletion parameters to assess nodal support and evolutionary relationships.

### Conserved motif and gene structure profiling

Conserved motifs in the EIN3/EIL proteins were predicted using the MEME suite v5.4.1 (Multiple EM for Motif Elicitation, https://meme-suite.org/meme/tools/meme), with parameters set to ZOOPS (zero or one occurrence per sequence), 15 motif discoveries, and motif length of 6–50 residues. Motif distributions were visualized using TBtools. Exon-intron architectures in *CoEIL* genes were extracted using *C. oleifera* genome annotation file [40] and mapped via the Exon-Intron Graphic Maker (http://www.wormweb.org/exonintron).

### Chromosomal location and promoter *Cis*-elements analysis

Chromosome lengths and *CoEIL* gene locations on the chromosomes were curated from the *C. oleifera* genome annotation file and plotted using MapGene2Chrom v2.1 (http://mg2c.iask.in/mg2c_v2.1/). Promoter regions (2000 bp upstream of transcription start sites) were screened for *cis*-elements using the PlantCARE database (https://bioinformatics.psb.ugent.be/webtools/plantcare/html/), with results visualized as a heatmap via MeV v4.9.

### Plant materials and experimental treatments

Eighteen 10-year-old *C. oleifera* (cv. ‘Cenruan 2’) trees cultivated at the Huadu Meilin Plantation (Guangzhou, China), were selected. Six months post-anthesis, trees were randomly assigned to three treatment groups (n = 6 per group): Ethephon treatment: 2.8 g L^-1^ ethephon (2-chloroethylphosphonic acid, an ethylene releaser) with 0.05% Tween 80 surfactant; Ethephon combined with brassinolide treatment: 2.8 g L^-1^ ethephon and 0.1 mg L^-1^ brassinolide (bioactive brassinosteroids) with 0.05% Tween 80; Control: aqueous 0.05% Tween 80 solution. For each tree, three branches (~ 30 fruits per branch) were tagged to quantify fruit abscission rates daily from day 0 (treatment day) to day 6 post-treatment. Remaining branches were harvested to collect abscission zone tissues, with each tree serving as a biological replicate. Abscission zone samples were dissected by excising 2-mm segments flanking the abscission plane, flash-frozen in liquid nitrogen, and stored at -80°C for downstream analyses.

### RNA extraction, cDNA synthesis, and *CoEIL* gene expression profiling

Total RNA was isolated from abscission zone tissues using the Column Plant RNAout 2.0 kit (TIANDZ, Beijing, China) following the manufacturer’s protocol. First-strand cDNA was synthesized from 1 µg RNA using Oligo (dT)_18_ primers and the TransScript One-Step gDNA Removal/ cDNA Synthesis SuperMix Kit (TransGen, Beijing, China). Quantitative real-time PCR was performed on a Bio-Rad CFX96 System (Hercules, USA) with Hieff qPCR SYBR Green Master Mix (Yeasen Biotech, Shanghai, China) to profile *CoEIL* gene expression. Thermal cycling conditions included: 95°C for 5 min (initial denaturation), 40 cycles of 95°C for 10 s, 55°C for 20 s, and 72°C for 30 s. The reference genes *CoTUB-α3* and *CoCESA* [48] were used for normalization, and relative expression was calculated by the 2^(-∆∆CT) method using an averaged CT-value from two reference genes. All reactions were conducted in triplicate, with primer sequences provided in S1 Table (Shanghai, China).

## Results

### Identification of four EIN3/EIL family members in *C. oleifera*

Homology-based screening of the *C. oleifera* genome using *A. thaliana* EIN3/EIL (AtEIL) amino acid sequences identified four CoEIL proteins, designated CoEIL1 to CoEIL4 (Table 1). These proteins range from 607 amino acid (aa) (CoEIL1) to 638 aa (CoEIL2), with nucleotide sequence lengths spanning 1,824 bp to 1,917 bp. Calculated molecular weights (MWs) varied between 68.84 kDa (CoEIL1) to 71.56 kDa (CoEIL2), while theoretical isoelectric points (pI) ranged from 5.22 (CoEIL1) to 6.33 (CoEIL3), confirming all CoEIL proteins as acidic (pI < 7.0). Biochemical characterization revealed intrinsic instability (instability index > 40) and hydrophilic properties (grand average of hydropathicity [GRAVY] = −0.677 to −0.875) across the family. Subcellular localization software (Cell-PLoc v2.0) predicted that all CoEIL proteins were present in the nucleus of the plant cells.

**Table 1:** Information on the EIN3/EIL family in *C. oleifera.*

Protein name	Gene ID	CDS (bp)	PL (aa)	MW (KDa)	pI	II	GRAVY	Sub	Chr	Location (bp)	
start	end
CoEIL1	PQ226491	1824	607	68.84	5.22	54.20	-0.753	Nucleus	12	81298871	81301029
CoEIL2	PQ226492	1917	638	71.56	5.82	57.68	-0.875	Nucleus	8	88017061	88018974
CoEIL3	PQ226493	1845	614	69.43	6.33	57.82	-0.837	Nucleus	6	111411514	111414505
CoEIL4	PQ226494	1875	624	70.39	5.43	52.47	-0.677	Nucleus	8	110059618	110061492

Note: CDS, coding sequence; PL, protein length; Mw, molecular weight; pI, isoelectric point; II, instability index; GRAVY, grand average of hydropathicity; Sub, subcellular localization; Chr, chromosome.

### Classification and phylogenetic analysis of CoEIL proteins

**Fig 1:**
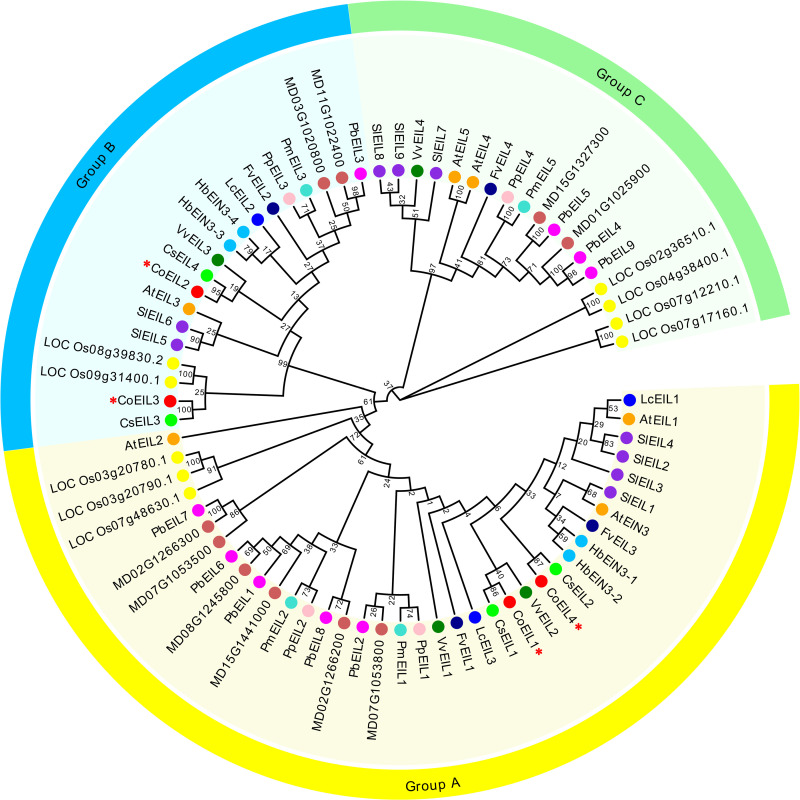
Phylogenetic analysis of plant EIN3/EIL proteins. The phylogenetic tree was constructed using the neighbor-joining method (1,000 bootstrap replicates; MEGA 7.0). Groups A-C were annotated with color-coded ribbons. Protein homologs from *Camellia oleifera* (Co, red), *Arabidopsis thaliana* (At, orange), *Prunus persica* (Pp, pink), *Vitis vinifer*a (Vv, green), *Litchi chinensis* (Lc, blue), *Solanum lycopersicum* (Sl, purple), *Malus × domestic*a (MD, chocolate), *Prunus mume* (Pm, cyan), *Pyrus bretschneideri* (Pb, magenta), *Fragaria vesca* (Fv, dark blue), *Hevea brasiliensis* (Hb, deep Sky Blue), *Camellia sinensist* (Cs, lime green), and *Oryza sativa* (LOC_Os, yellow) are denoted by coded circles. CoEIL proteins are specifically designated by red star symbols (★). Detailed protein information is provided in S2 Table.

To elucidate phylogenetic relationships among EIN3/EIL transcription factors across plant species, we performed multiple sequence alignment and phylogenetic reconstruction using 74 protein sequences, including four CoEIL proteins from *C. oleifera* and 70 homologs from 12 representative plant species, such as *A. thaliana*, peach, grape, litchi, tomato, apple, mei (*Prunus mume*), pear (*Pyrus bretschneideri*), strawberry (*Fragaria vesca*), rubber tree, tea (*Camellia sinensis*), and rice. Phylogenetic analysis resolved three distinct clades (Fig 1): Group A (36 members), Group B (20 members), and Group C (18 members). CoEIL1 and CoEIL4 clustered within Group A, whereas CoEIL2 and CoEIL3 formed a subgroup in Group B. Notably, CoEIL1 exhibited high sequence homology with CsEIL1 (tea), VvEIL2 (grape), and LcEIL3 (litchi), while CoEIL2 showed evolutionary proximity to CsEIL4 (tea) and VvEIL3 (grape). Comparative analysis further revealed conserved domain sharing between CoEIL3/CoEIL4 and CsEIL3/CsEIL2 in tea, respectively.

### Multiple sequence alignment and conserved motif analysis of CoEIL proteins

**Fig 2:**
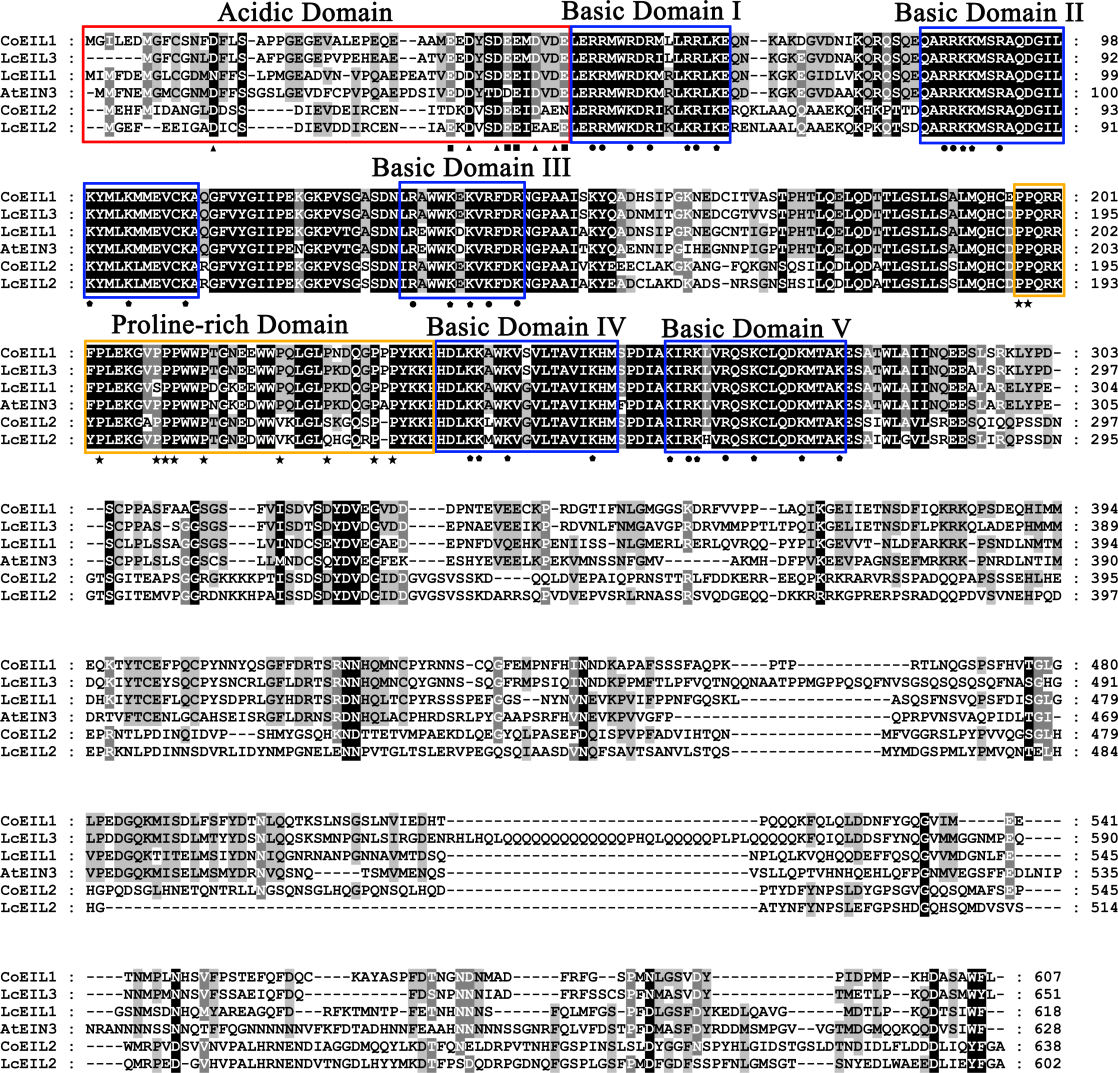
Sequence alignment of EIN3/EIL proteins from *C. oleifera* (Co), *A. thaliana* (At), and litchi (Lc). Multiple sequences alignment was performed using ClustalX software. Residues exhibiting 100% identity are highlighted in black, while regions with > 80% and > 60% sequence identity are marked in dark-grey and light-grey shading, respectively. The acidic domain is delineated by a red rectangle, the proline-rich domain by an orange rectangle, and the five basic domains by blue rectangles. Distinct geometric symbols denote specific amino acids: triangles (△) for aspartic acid, squares (□) for glutamic acid, circles (○) for arginine, pentagons (⬠) for lysine, and stars (★) for proline.

Multiple sequence alignments of CoEIL proteins with EIN3/EIL homologs from *A. thaliana* (AtEIN3) and litchi (LcEIL1, LcEIL2, and LcEIL3) revealed strong N-terminal conservation and divergent C-terminal regions (Fig 2). Structural analysis identified seven conserved domains including an acidic domain enriched in aspartic acid and glutamic acid residues and five basic domains (I-V) characterized by arginine and lysine conservation. A proline-rich region exhibited near-complete sequence conservation.

**Fig 3:**
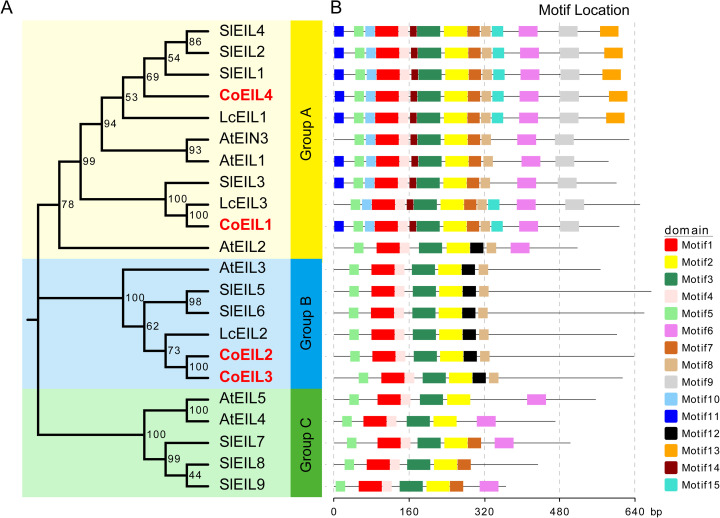
Conserved motif analysis of plant EIN3/EIL proteins. (A) Phylogenetic tree constructed using the Neighbor-Joining method (MEGA 7.0; using 1,000 bootstrap replicates. (B) Distribution of 15 conserved motifs identified via MEME suite. Motifs are represented by color-coded boxes, with lengths and positions scaled proportionally to protein sequences (bottom ruler).

MEME-based motif discovery (Fig 3 and S1 Fig) delineated 15 conserved motifs, six of which overlapped with functional domains characteristic of plant EIL proteins. Motif 5 was associated with domain basic domain I, and motif 1 was related to domain basic domain II. Motif 4 contained domain basic domain III, and motif 2 contained domains basic domain IV and V. The proline-rich region contained motifs 2 and 3. The acidic domain contained motifs 6 and 11. Most EIN3/EIL proteins contained motifs 1, 2, 3, 4, and 5. Notably, phylogenetically related EIN3/EIL proteins within the same clade shared conserved motif architectures. Group A members (CoEIL1 and CoEIL4) harbored unique motifs (motifs7, 9, 10, 11, 13, 14, 15), while Group B (CoEIL2 and CoEIL3) exclusively contained motif 12.

### Chromosom al localization and exon-intron architecture of *CoEIL* genes

**Fig 4:**
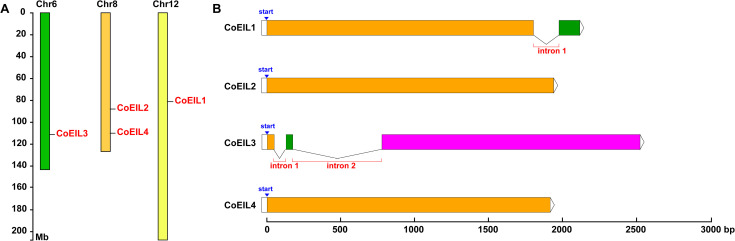
Chromosome localization and exon-intron architecture of *CoEIL* genes in *C. oleifera.* (A) Chromosomal distribution of *CoEIL* localization. Chromosome numbers are labeled above each bar. The scale bar (left) denotes physical distances (megabase, Mb). Genomic coordinates for *CoEIL* positions are provided in Table 1. (B) Exon-intron structures of *CoEIL* genes. Exons (color boxes), introns (black lines), and the untranslated regions in the two terminals (UTRs; white boxes) are illustrated with proportional lengths.

Four *CoEIL* genes were mapped to three chromosomes (6, 8, and 12) in *C. oleifera* genome (Fig 4A). Chromosome 8 harbored two loci (*CoEIL2* and *CoEIL4*), while chromosomes 6 and 12 each contained a single *CoEIL* locus (*CoEIL1* on chromosome 12 and *CoEIL3* on chromosome 6), indicating non-uniform chromosomal distribution. Exon-intron structural analysis revealed divergence among *CoEIL* family members (Fig 4B): *CoEIL3* exhibited a bifurcated structure with two introns, *CoEIL1* contained a single intron, and the remaining *CoEIL* genes (*CoEIL2* and *CoEIL4*) lacked introns entirely, consisting of uninterrupted coding sequences. This structural heterogeneity suggests intron-mediated regulatory evolution.

### *Cis*-acting regulatory elements in *CoEIL* promoters

**Fig 5:**
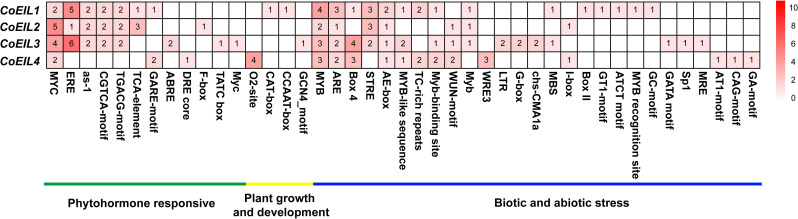
*Cis*-acting regulatory elements in *CoEIL* promoter regions. Color-coded grids denote the quantity of responsive elements identified in each *CoEIL* gene.

To identify putative regulatory motifs, we analyzed 2.0 kb promotor regions upstream of *CoEIL* genes for hormone-responsive, growth-related, and stress-associated *cis*-elements (Fig 5). A total of 12 hormone-responsive, 4 developmental, and 27 stress-linked regulatory motifs were annotated (S3 Table). Individual *CoEIL* promoters harbored 5–20 hormone*-*responsive and 10–27 stress-associated elements. Core regulatory motifs conserved across four *CoEIL* genes included MYC (methyl jasmonate responsiveness), MYB (MYB transcription factor binding sites), ARE (anaerobic response), and AE-box (light responsiveness). Three *CoEIL* paralogs shared additional elements: ethylene-responsive (ERE), methyl jasmonate-responsive (as-1, CGTCA-motif, and TGACG-motif), light-responsive (Box 4), stress response (STRE), MYB-binding (MYB-like and Myb-binding site), and wounding-response (WUN-motif) sequences. These findings suggest *CoEIL* genes are transcriptionally regulated by diverse hormonal and environmental signals.

### Effects of exogenous brassinosterid and ethephon on fruit abscission and *CoEIL* genes expression in *C. oleifera*

**Fig 6:**
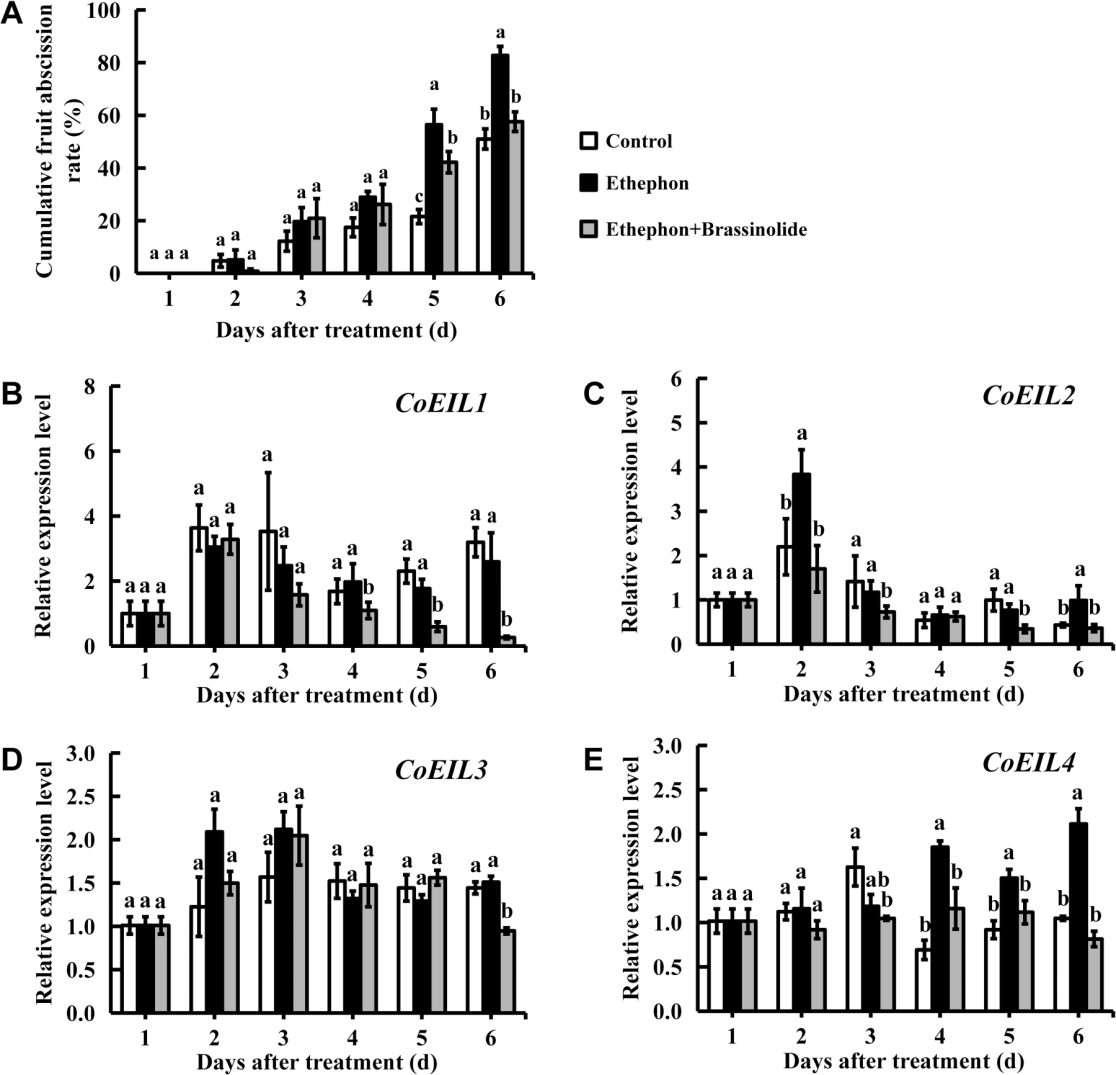
Effects of brassinolide and ethephon on fruit abscission and *CoEIL* genes expression in *C. oleifera.* (A) Cumulative abscission rate of fruitless. (B-E) Quantitative real-time PCR analysis of *CoEIL* genes expression levels in fruit abscission zone. Letters denote significant differences (Duncan’s multiple range test, p < 0.05).

Ethylene and brassinosteroids are known as antagonists in the regulation of fruit abscission in some plant species. To assess hormonal regulation of fruit abscission, *C. oleifera* fruits were treated with ethephon (ethylene generator) or ethephon combined with brassinolide. As shown in Fig 6A, ethephon significantly accelerated fruit drop, reaching 82% cumulative fruit abscission at day 6 post-treatment, compared to 51% in controls. Co-application of brassinolide and ethephon mitigated this effect, reducing abscission to 57% at day 6 post-treatment.

Quantitative real-time PCR revealed ethephon- and brassinolide-responsive *CoEIL* expression patterns in abscission zone (Fig 6B-E). Ethephon significantly induced *CoEIL2* expression in abscission zone tissues, with 1.74-fold and 2.29-fold increases relative to controls at day 4 and 6 post-treatment, respectively (Fig 6C). Similarly, *CoEIL4* expression increased to 2.68-fold (day 4 post-treatment), 1.63-fold (day 5 post-treatment), and 2.02-fold (day 6 post-treatment), respectively (Fig 6E). In contrast, co-application of brassinolide and ethephon suppressed *CoEIL1* and *CoEIL4* expression at day 4–6 post-treatment (Fig 6B, E), and reduced *CoEIL2* expression by 55.63% (day 2 post-treatment), 37.96% (day 4 post-treatment), 55.19% (day 5 post-treatment), and 63.29% (day 6 post-treatment) compared to ethephon alone, respectively (Fig 6C). However, *CoEIL1* gene expression showed no significant differences between ethylene-treated and control groups. And *CoEIL3* expression exhibited minimal variation across the three experimental treatments (Fig 6D).

## Discussion

The EIN3/EIL gene family, pivotal to ethylene signaling and plant development [49], is widely conserved across economically significant crops [50]. Here, we report the first genome-wide identification of *EIN3/EIL* homologs in *C. oleifera*, revealing four *CoEIL* genes (Table 1). This count is lower than in rice (9; *OsEIL* genes) [27], *A. thaliana* (6; *AtEIL* genes) [19], Zea mays (9; *ZmEIL* genes) [51], pear (10; *SlEIL* genes) [52], broomcorn millet (15; *PmEIL* genes) [53], and bread wheat (21; *TaEIL* genes) [54], but comparable to peaches, grapes, rubber trees, and strawberries [28,52,55], suggesting no direct correlation between *EIN3/EIL* copy number and genome size [52,54]. All CoEIL proteins are acidic (pI < 7) and putatively nuclear-localized (Table 1), consistent with their roles as transcription factors [54].

Phylogenetic analysis of EIN3/EIL proteins from 13 species (including *C. oleifera*, *A. thaliana*, peach, grape, litchi, tomato, apple, mei, pear, strawberry, rubber tree, tea, and rice) resolved three clades (Group A, B, and C) (Fig 1). The interspersed monocot-dicot membership, across clades supports diversification prior to angiosperm divergence [50]. Structurally, CoEIL proteins retain conserved N-terminal domain (containing an acidic region), five basic domains (I-V), and proline-rich regions (PR) critical for DNA binding [19,56], while exhibiting divergent C-terminal sequences (Fig 2). Motif architecture analysis (Fig 3) revealed clade-specific signatures. Group A harbored seven unique motifs (7, 9, 10, 11, 13, 14, and 15), whereas Group B retained motif 12. This divergence suggests functional specialization, warranting further investigation into CoEIL roles in ethylene-mediated processes.

Promoter analysis of *CoEIL* genes revealed conserved hormone-responsive *cis*-elements, including methyl jasmonate-, ethylene-, gibberellin-, abscisic acid-, and brassinosteroid-associated motifs (Fig 5). All *CoEIL* promoters harbored methyl jasmonate-response elements. Exogenous jasmonic acid or its derivative methyl jasmonate, as an activator in organ abscission, has been reported [57–59]. Notably, *coi1* (CORONATINE INSENSITIVE1, a jasmonic acid receptor) mutants in *A. thaliana* demonstrate jasmonic acid’s ethylene-independent regulation of floral organ abscission [60], suggesting parallel pathways in *C. oleifera*. Besides jasmonic acid, the action and concomitant hormonal imbalance of ethylene, abscisic acid, brassinosteroids, gibberellins, and auxin in organ abscission have been reported [40,61–64]. Ethylene response elements (ERE) were identified in *CoEIL1*, *CoEIL2*, and *CoEIL3*, but not *CoEIL4*, implicating that three paralogs may be ethylene-inducible (Fig 5). Gibberellins-response elements (GARE-motif, F-box, TATC box) were ubiquitous across *CoEIL* promoters, while abscisic acid-response elements (ABRE, DRE core) localized exclusively to *CoEIL3* and *CoEIL4*, indicating differing sensitivities to phytohormone factors. Brassinosteroids-response element (BRRE; CGTGC/TG) were detected in *CoEIL1* and *CoEIL2,* with E-box element (CANNTG) present in all *CoEIL* genes (4–9 copies/gene), a group of *cis*-acting element identified from promoter analysis of many brassinosteroids-regulated genes in plants [65–66]. These findings suggest *CoEIL* genes are transcriptionally regulated by synergistic or antagonistic hormone networks. Additionally, stress-responsive *cis*-elements in *CoEIL* genes*’* promoters imply roles in biotic/abiotic stresses adaptation, warranting functional validation.

EIN3/EIL transcription factors are established regulators of organ abscission across plant species. In tomato, suppression of *LeEIL* genes reduces ethylene sensitivity and delays floral organ abscission [22], while in litchi, *LcEIL2/3* drives ethylene-induced fruitlet abscission via ethylene biosynthesis and cell wall remodeling genes [21]. Similarly, CISPR-Cas9 knockout of *EIL3*, *EIL4*, and *EIN2L* in soybean (*Glycine max*) increases yield by 65% [67]. We hypothesized that ethylene-induced fruit abscission in *C. oleifera* can be mitigated by modulating ethylene biosynthesis and signaling pathways. To test this, we compared three treatments: control (baseline abscission), ethephon (ethylene-induced abscission), and ethephon + brassinolide (co-application of ethephon and brassinolide; brassinolide antagonism-inhibit abscission). Paradoxically, quantitative real-time PCR analysis revealed no significant correlation between *CoEIL1* to *CoEIL4* expression in the abscission zone and abscission rates. Despite containing >5 ethylene-response elements (EREs) in their promoters, *CoEIL1* and *CoEIL3* exhibited non-significant ethylene responsiveness (fold change < 2), but were strongly suppressed under co-application of brassinolide and ethephon, suggesting sensitivity to brassinolide response or alternative regulatory mechanisms. In contrast, *CoEIL2* (1 ERE element in promoter) and *CoEIL4* (no ERE element in promoter) showed moderate ethylene induction with delayed and inconsistent expression kinetics, implying involvement of non-canonical cis-elements (e.g., E-box elements). Co-application of brassinolide and ethephon suppressed all *CoEIL* genes expect *CoEIL3*, possibly via BRRE or E-box elements, consistent with *A. thaliana* studies showing BZR1 (BRASSINOZALE- RESISTANT1) and EIN3 interaction in brassinosteroid-ethylene crosstalk [68]. These findings suggest ethylene signaling in *C. oleifera* abscission zones may involve extra-fruit ethylene production or tissue-specific signal transduction pathways distinct from characterized *CoEIL* regulatory networks. The observed changes during abscission likely originate from ethylene-induced senescence and blocked polar auxin transport in fruits [69], where the expression levels of *EIL* genes may exhibit more pronounced dynamics.

## Conclusion

In conclusion, this study delivers a comprehensive characterization of the *EIN3/EIL* gene family in the *C. oleifera* genome, elucidating phylogenetic relationships, conserved domains architectures, exon-intron distributions, chromosomal localization, *cis-*regulatory elements, and hormone-responsive expression dynamics. We identified four *CoEIL* genes phylogenetically grouped into Group A and Group B, all retaining hallmark structural features of plant EIN3/EIL proteins, including conserved N-terminal domains and variable C-terminal regions. Although quantitative real-time PCR analysis in abscission zone tissues revealed dynamic expression patterns of *CoEIL* genes, no significant correlation was observed between their transcript levels and fruit abscission rates, suggesting that canonical EIN3/EIL-mediated abscission mechanisms may operate beyond the fruit abscission zones itself.

## Supporting information

S1 Table Primer sequences of the genes used in this research. (XLSX)

S2 Table List of plant EIN3/EIL proteins used in this study. (XLSX)

S3 Table All *Cis-*elements in 2.0 kb upstream region of *CoEIL* genes. (XLSX)

S1 Fig Highly conserved motifs identified in EIN3/EIL proteins. Sequence logos were based on full-length alignment using MEME analysis. The bit score indicates the information content of each position in the sequence.
(DOCX)
