# Supplementary material for: Genome-wide identification and characterization of Ethylene-Insensitive 3 (EIN3/EIL) gene family in Camellia oleifera
Source: PLoS One. 2025 May 23;20(5):e0324651. doi: 10.1371/journal.pone.0324651 (PMC12101669; doi:10.1371/journal.pone.0324651)
Supplement: S1 Fig — Sequence logos were based on full-length alignment using MEME analysis. The bit score indicates the information content of each position in the sequence. (DOCX) [file pone.0324651.s004.docx]

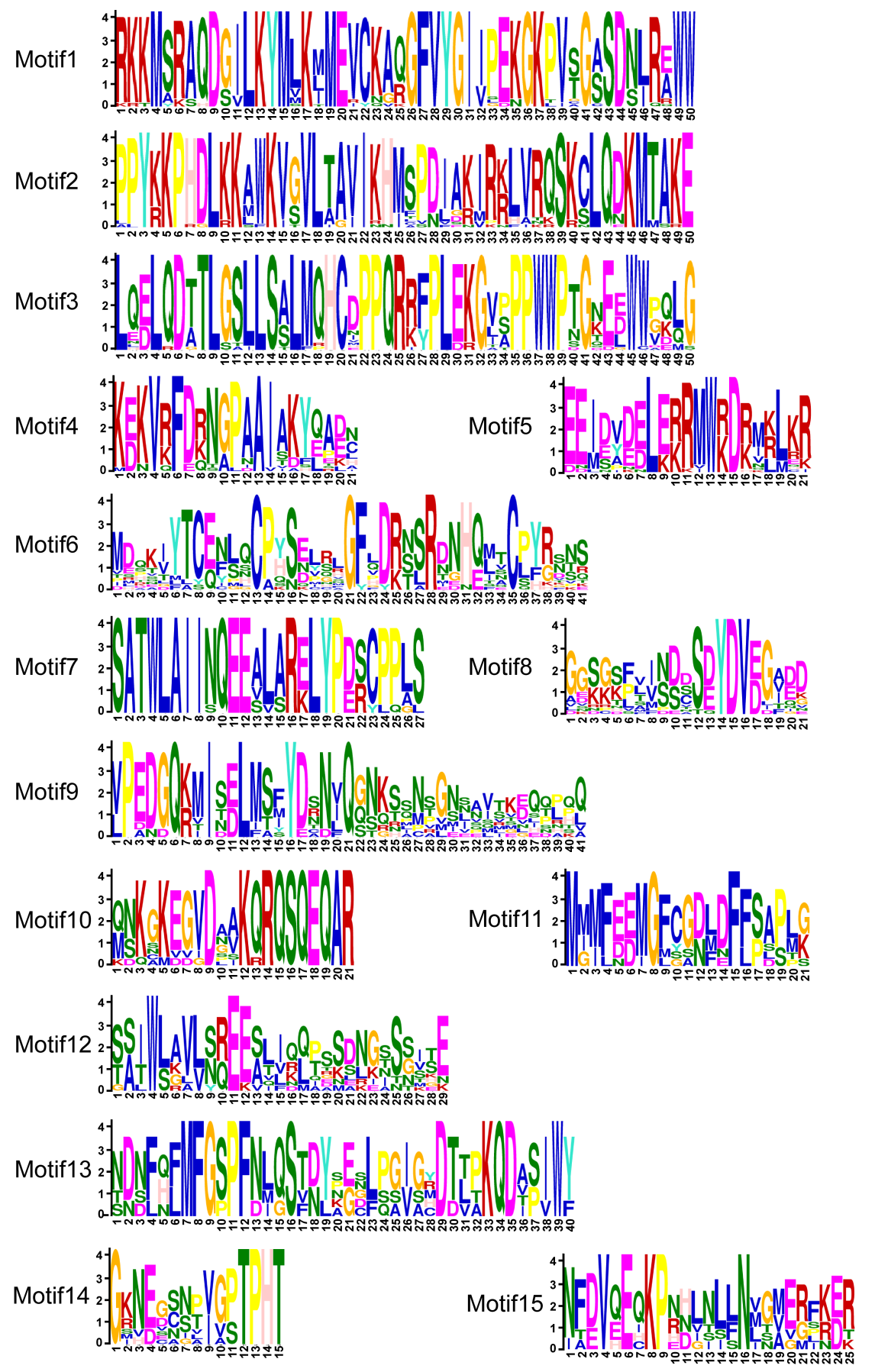


**S1 Fig. Highly conserved motifs identified in EIN3/EIL proteins.** Sequence logos were based on full-length alignment using MEME analysis. The bit score indicates the information content of each position in the sequence.
